# Genetic and Environmental Factors Contributing to Cardiovascular Malformation: A Unified Approach to Risk

**DOI:** 10.1161/JAHA.113.000292

**Published:** 2013-06-21

**Authors:** Robert B. Hinton

**Affiliations:** 1 The Heart Institute, Division of Cardiology, Cincinnati Children's Hospital Medical Center, Cincinnati, OH (R.B.H.)

**Keywords:** editorials, genetics, environment, risk factors, congenital heart disease

## Introduction

In the late 19th century, William Osler notoriously quipped that treating infants with cardiac defects was futile, thereby encouraging Maude Abbott to pursue her rigorous studies of pathologic heart specimens. Ultimately, this work resulted in *The Atlas of Congenital Cardiac Disease*, which is widely regarded as the first systematic catalogue of cardiovascular malformation (CVM) and therefore the first classification system for clinical pediatric cardiology. Organizing malformations requires consideration of cause. The investigations of the etiology of CVM, also known as congenital heart disease (CHD), has advanced on 2 largely independent lines of thought: environmental or genetic.^1–2^ During the same time, the advent and rapid evolution of surgical intervention for these conditions defined an exquisite clinical taxonomy that was based on anatomy and physiology.^3^ Only recently have there been efforts to reconcile environmental and genetic factors in a clinically meaningful manner and integrate this information into the existing classification scheme.

Classification schemes for CVM continue to present significant challenges for both clinicians and researchers. When considering cause, it is important to group lesions appropriately and avoid misclassification that may confound interpretation. To this end, classification paradigms have been developed that account for the developmental relationships of lesions, in addition to the anatomic relationships previously described, to increase confidence in grouping.^4^ The National Birth Defects Prevention Study developed an exhaustive taxonomy that organizes CVM in multiple ways, including the most specific definition of a single defect (eg, hypoplastic left heart syndrome—the so‐called “splitting” approach), the most broad groupings (eg, left sided outflow tract obstruction lesions—the “clumping” approach), and an intermediate level that allows flexibility with the analysis of common associations (eg, aortic stenosis and coarctation of the aorta).^5^ Overall, a classification system that incorporates etiologic factors as well as deep phenotyping is necessary for clinical and research advances alike.

In this issue of JAHA: *Journal of the American Heart Association*, Fung et al^6^ have made an important step toward unifying the analysis of environmental and genetic risk factors for CVM. Importantly, they have called for increased surveillance that includes a more rigorous ascertainment of risk factors in general and comprehensive integration of different types of CVM risk in particular. An important strength of the study is the use of the International Nomenclature for Congenital Heart Surgery that utilizes developmentally informed splitting and clumping approaches. This study reports both genetic risk factors (family history, genetic testing results, and the presence of extra‐cardiac anomalies) and environmental risk factors (maternal health, maternal exposures, and pregnancy complications) in over 2300 pediatric patients with CVM in the present era. This approach combines exhaustive information from divergent perspectives, as opposed to an assessment of only one or the other, and analyzes genetic and environmental risk factors together, recognizing that elucidating the genetic basis of CVM will require a thoughtful assessment of environmental factors that can influence disease causation.

In the age of genetics, environmental factors often are not acknowledged or recognized, or are viewed as noncontributory or secondary. However, the Baltimore Washington Infant Study (BWIS) and others have reported clear associations between CVM and a variety of environmental factors, including diabetes and retinoic acid.^7^ Prenatal risk factors are broadly defined as something that increases the chances of developing a disease. Risk factors contribute to the manifestation of disease; they may or may not cause disease. In the context of this study, clinical risk factors for CVM have been grouped as maternal health (age, prepregnancy BMI, type 1 diabetes status), maternal exposures (smoking, medications, chemicals), and complications of pregnancy (hypertension, infection, gestational diabetes). Among several positive findings, some of which confirm established associations, some are either new or of general interest. For example, the association between increased paternal age and CVM was only seen in children with genetic abnormalities, suggesting the environmental factor is a risk for genetic abnormalities that may include CVM, but in the absence of a genetic abnormality may not pose an additional risk. Interestingly, in the present study, smoking during early pregnancy was associated with an increased risk of CVM (OR 2.0 [1.2 to 3.5], *P*=0.0004), the relative risk consistent with previous studies. Since smoking is common and modifiable, it is a good target for intervention,^8^ but causality has not been established like it has for smoking's adverse impact on fetal growth, in part due to several studies that have shown no association. Interestingly, it was noted that there has been a significant decrease in maternal smoking recently, suggesting it may be possible to identify the impact of smoking cessation on CVM in the near future.

Environmental risk factors include over‐the‐counter, prescription, and fertility medications. The sensational example of thalidomide was followed by the evaluation of numerous medications as potential causes of CVM, including recently bupropion,^9^ which resulted in the significant reclassification of the medication's pregnancy risk profile from Category C to Category D by the Food and Drug Administration. Weak associations are difficult to interpret because they may represent true risk or false association. Interpreting epidemiologic data requires an understanding of the strengths and weaknesses of the case–control study design. Most importantly, association does not prove causation, and spurious associations may result for a number of reasons, including recall bias, resulting in conflicting or inconsistent findings. The current study's observation that maternal drug use during pregnancy is increasing underscores the importance of understanding the safety of commonly used drugs.

There have been instances where a drug is considered a risk based on early epidemiologic evidence and that risk is later determined to be incorrect, raising questions about what the level of proof should be in this context. For example, lithium was shown to be associated with CVM, in particular Ebstein anomaly of the tricuspid valve, but after more analyses this collective position was reversed. So, while the possibility of lithium teratogenicity has not been excluded, the inconsistency in results undermined the confidence in a possible small risk and ultimately became a cautionary tale for association studies in pediatric cardiology.^10–11^ Bradford Hill reported a seminal approach to examining causation in the context of association that includes a rigorous assessment of several criteria, including strength of association, temporality, consistency, biological plausibility, coherence and analogy, a dose‐response, and experimental support of mechanism.^12^ These considerations will be helpful in the ongoing assessment of the potential adverse effects of specific medications that may be associated with CVM.

Fung et al described several interesting observations pertaining to genetic risk factors. Importantly, the family history was demonstrated to be the most reliable tool in establishing risk. Genetic testing increased significantly from 9% to 25% from 1990 to 2011, and while the overall yield was only 10%, if there was a positive family history or the presence of an extracardiac anomaly, then the yield increased to 32% and 43%, respectively. Recently, genetic testing patterns between geneticists and cardiologists were explored in a cohort of CVM patients, identifying inconsistencies about perceived indications for testing and the need for a systematic approach clinically as well as larger, more robust research studies that combine careful phenotyping with genetic and environmental factors.^13^ Taken together, these observations emphasize the fact that while there is significant evidence that most CVM has a genetic component, we are only able to identify a small proportion of genetic causes presently.

Identifiable single‐gene mutations account for a small minority of cases and indications to screen specific genes are limited. As more causes are identified and sequencing technology advances, the ability to identify complex or polygenic disorders like nonsyndromic sporadic CVM will improve. In addition, these technologies will provide insight into gene–gene and gene–environment interactions. Increasingly, networks of genetic modifiers will be elucidated that better define genetic risk. For example, the Pediatric Heart Network identified single nucleotide polymorphisms (SNPs) in 5 genes within the Renin–Angiotensin–Aldosterone system that predict failure of normal postoperative remodeling as well as poor somatic growth, suggesting that the presence of these variants may predict adverse outcome and therefore identify a subset of patients at relatively high risk.^14^ This approach on a broad scale may resemble a comprehensive risk profile for all types of CVM, much like newborn screening. Advances in personalized medicine are already leveraging the clinical utility of genetic information to identify specific risks in specific people,^15^ suggesting that we are quickly approaching a paradigm shift in preventive management.

There is ample evidence that nonsyndromic CVM is polygenic, and gene–gene and gene–environment interactions will play a significant role in pathogenesis.^16–18^ The involvement of multiple factors presents challenges for assessing risk and defining predisposition. Classically, a genetic predisposition involving one or more genetic abnormalities is followed by an environmental insult where both types of factors are required for disease manifestation. Little is known at present about how this paradigm might be applied to CVM. Accordingly, the authors call for a more rigorous ascertainment of both genetic and environmental risk factors, including gene–environment interactions that contribute to CVM. This might translate to a systematic approach that incorporates all of these factors in electronic medical record and data registries for clinical use and multisite research. As more is learned about the genetic basis of CVM and the impact of environmental factors, composite risk profiles that capture both genetic and environmental factors may allow the development of clinically useful thresholds. As the authors explain, these advances will facilitate our ability to implement prevention and early risk stratification and prevention strategies through improved diagnostic testing, as well as more effective early intervention and targeted therapeutic plans through improved patient‐specific risk definition.

## References

[1] Jenkins KJ Correa A Feinstein JA Botto L Britt AE Daniels SR Elixson M Warnes CA Webb CL American Heart Association Council on Cardiovascular Disease in the Young Noninherited risk factors and congenital cardiovascular defects: current knowledge: a scientific statement from the American Heart Association Council on Cardiovascular Disease in the Young: endorsed by the American Academy of Pediatrics. Circulation. 2007; 115:2995-3014 17519397 10.1161/CIRCULATIONAHA.106.183216

[2] Pierpont ME Basson CT Benson DW Jr Gelb BD Giglia TM Goldmuntz E McGee G Sable CA Srivastava D Webb CL American Heart Association Congenital Cardiac Defects Committee, Council on Cardiovascular Disease in the Young Genetic basis for congenital heart defects: current knowledge: a scientific statement from the American Heart Association Congenital Cardiac Defects Committee, Council on Cardiovascular Disease in the Young: endorsed by the American Academy of Pediatrics. Circulation. 2007; 115:3015-3038 17519398 10.1161/CIRCULATIONAHA.106.183056

[3] Fyler DC Rudolph AM Wittenborg MH Nadas AS Ventricular septal defect in infants and children; a correlation of clinical, physiologic, and autopsy data. Circulation. 1958; 18:833-851 13585559 10.1161/01.cir.18.5.833

[4] Clark EB Pathogenetic mechanisms of congenital cardiovascular malformations revisited. Semin Perinatol. 1996; 20:465-472 9090774 10.1016/s0146-0005(96)80062-0

[5] Botto LD Lin AE Riehle‐Colarusso T Malik S Correa A National Birth Defects Prevention Study Seeking causes: classifying and evaluating congenital heart defects in etiologic studies. Birth Defects Res A Clin Mol Teratol. 2007; 79:714-727 17729292 10.1002/bdra.20403

[6] Fung A Manlhiot C Naik S Rosenberg H Smythe J Lougheed J Mondal T Chitayat D McCrindle B Mital S Impact of prenatal risk factors on congenital heart disease in the current era. J Am Heart Assoc. 2013; 2:e00006410.1161/JAHA.113.000064 23727699 10.1161/JAHA.113.000064 PMC3698764

[7] Ferencz C Loffredo CA Correa‐Villasenor A Wilson PD Genetic and Environmental Risk Factors for Major Congenital Heart Disease: The Baltimore Washington Infant Study 1981–1989. 1997 Mount Kisco, NY Futura Publishing

[8] Malik S Cleves MA Honein MA Romitti PA Botto LD Yang S Hobbs CA National Birth Defects Prevention Study Maternal smoking and congenital heart defects. Pediatrics. 2008; 121:e810-e816 18381510 10.1542/peds.2007-1519

[9] Alwan S Reefhuis J Botto LD Rasmussen SA Correa A Friedman JM National Birth Defects Prevention Study Maternal use of bupropion and risk for congenital heart defects. Am J Obstet Gynecol. 2010; 203:52.e1-52.e6 20417496 10.1016/j.ajog.2010.02.015

[10] Jacobson SJ Jones K Johnson K Ceolin L Kaur P Sahn D Donnenfeld AE Rieder M Santelli R Smythe J Prospective multicentre study of pregnancy outcome after lithium exposure during first trimester. Lancet. 1992; 339:530-533 1346886 10.1016/0140-6736(92)90346-5

[11] Cohen LS Friedman JM Jefferson JW Johnson EM Weiner ML A reevaluation of risk of in utero exposure to lithium. JAMA. 1994; 271:146-150 8031346

[12] Hill AB The environment and disease: association or causation? Proc R Soc Med. 1965; 58:295-300 14283879 10.1177/003591576505800503 PMC1898525

[13] Connor JA Hinton RB Miller EM Sund KL Ruschman JG Ware SM Genetic testing practices in infants with congenital heart disease. Con Heart Dis. 2013 10.1111/chd.12112 23782710

[14] Mital S Chung WK Colan SD Sleeper LA Manlhiot C Arrington CB Cnota JF Graham EM Mitchell ME Goldmuntz E Li JS Levine JC Lee TM Margossian R Hsu DT Pediatric Heart Network Investigators Renin‐angiotensin‐aldosterone genotype influences ventricular remodeling in infants with single ventricle. Circulation. 2011; 123:2353-2362 21576655 10.1161/CIRCULATIONAHA.110.004341 PMC3137902

[15] Pulley JM Denny JC Peterson JF Bernard GR Vnencak‐Jones CL Ramirez AH Delaney JT Bowton E Brothers K Johnson K Crawford DC Schildcrout J Masys DR Dilks HH Wilke RA Clayton EW Shultz E Laposata M McPherson J Jirjis JN Roden DM Operational implementation of prospective genotyping for personalized medicine: the design of the Vanderbilt PREDICT project. Clin Pharmacol Ther. 2012; 92:87-95 22588608 10.1038/clpt.2011.371 PMC3581305

[16] Chang CP Bruneau BG Epigenetics and cardiovascular development. Annu Rev Physiol. 2012; 74:41-68 22035349 10.1146/annurev-physiol-020911-153242

[17] Obermann‐Borst SA Isaacs A Younes Z van Schaik RH van der Heiden IP van Duyn CM Steegers EA Steegers‐Theunissen RP General maternal medication use, folic acid, the MDR1 C3435T polymorphism, and the risk of a child with a congenital heart defect. Am J Obstet Gynecol. 2011; 204:236.e1-236.e8 21183151 10.1016/j.ajog.2010.10.911

[18] Zaidi S Choi M Wakimoto H Ma L Jiang J Overton JD Romano‐Adesman A Bjornson RD Breitbart RE Brown KK Carriero NJ Cheung YH Deanfield J Depalma S Fakhro KA Glessner J Hakonarson H Italia MJ Kaltman JR Kaski J Kim R Kline JK Lee T Leipzig J Lopez A Mane SM Mitchell LE Newburger JW Parfenov M Pe'er I Porter G Roberts AE Sachidanandam R Sanders SJ Seiden HS State MW Subramanian S Tikhonova IR Wang W Warburton D White PS Williams IA Zhao H Seidman JG Brueckner M Chung WK Gelb BD Goldmuntz E Seidman CE Lifton RP De novo mutations in histone‐modifying genes in congenital heart disease. Nature. 2013; ???:???-??? 23665959 10.1038/nature12141 PMC3706629

